# End-to-End Training for Compound Expression Recognition

**DOI:** 10.3390/s20174727

**Published:** 2020-08-21

**Authors:** Hongfei Li, Qing Li

**Affiliations:** 1 Institute of Microelectronics of Chinese Academy of Sciences, Beijing 100029, China; lihongfei@ime.ac.cn; 2 University of Chinese Academy of Sciences, Beijing 100049, China

**Keywords:** joint training, end-to-end, deep SAE, compound expression, appearance feature, geometric feature, model ensembling, frequency domain transform, stacked LSTM, Sequence-to-Sequence

## Abstract

For a long time, expressions have been something that human beings are proud of. That is an essential difference between us and machines. With the development of computers, we are more eager to develop communication between humans and machines, especially communication with emotions. The emotional growth of computers is similar to the growth process of each of us, starting with a natural, intimate, and vivid interaction by observing and discerning emotions. Since the basic emotions, angry, disgusted, fearful, happy, neutral, sad and surprised are put forward, there are many researches based on basic emotions at present, but few on compound emotions. However, in real life, people’s emotions are complex. Single expressions cannot fully and accurately show people’s inner emotional changes, thus, exploration of compound expression recognition is very essential to daily life. In this paper, we recommend a scheme of combining spatial and frequency domain transform to implement end-to-end joint training based on model ensembling between models for appearance and geometric representations learning for the recognition of compound expressions in the wild. We are mainly devoted to digging the appearance and geometric information based on deep learning models. For appearance feature acquisition, we adopt the idea of transfer learning, introducing the ResNet50 model pretrained on VGGFace2 for face recognition to implement the fine-tuning process. Here, we try and compare two minds, one is that we utilize two static expression databases FER2013 and RAF Basic for basic emotion recognition to fine tune, the other is that we fine tune the model on the input three channels composed of images generated by DWT2 and WAVEDEC2 wavelet transforms based on rbio3.1 and sym1 wavelet bases respectively. For geometric feature acquisition, we firstly introduce a densesift operator to extract facial key points and their histogram descriptions. After that, we introduce deep SAE with a softmax function, stacked LSTM and Sequence-to-Sequence with stacked LSTM and define their structures by ourselves. Then, we feed the salient key points and their descriptions into three models to train respectively and compare their performances. When the model training for appearance and geometric features learning is completed, we combine the two models with category labels to achieve further end-to-end joint training, considering that ensembling models, which describe different information, can further improve recognition results. Finally, we validate the performance of our proposed framework on an RAF Compound database and achieve a recognition rate of 66.97%. Experiments show that integrating different models, which express different information, and achieving end-to-end training can quickly and effectively improve the performance of the recognition.

## 1. Introduction

Human language is divided into natural language and body language. Facial expression is part of body language. As a non-linguistic signal of human beings [1], facial expressions contain rich personal information, social interaction information, and also convey some information about people’s cognitive behavior, temperament, personality, authenticity, psychology, and almost all of that information cannot be replaced by other means of information expression. Therefore, accurately identifying the expression of others is critical to successful human–computer interaction. When people see different people’s faces, they can easily recognize the same expression, which is called facial expression recognition.

The study of facial expression began in the 19th century. In 1872, Darwin elaborated on the connection and difference between human facial expression and animal facial expression in his famous work [2]. In 1971, Ekman and Friesen did pioneering work on modern facial expression recognition [3]. They studied six basic human expressions (i.e., happy, sad, surprised, fearful, angry, disgusted), determining the category of objects to be identified, systematically established a facial expression database with thousands of different samples, and described the corresponding facial changes of each expression in detail, including how eyebrows, eyes, eyelids, lips change, and so on. In 1978, Suwa et al. [4] carried out the first attempt of facial expression recognition on a video animation of faces, and proposed automatic facial expression analysis on the image sequences. Since the 1990s, K. mase [5] have used optical flow to determine the main direction of muscle movement. After using the proposed optical flow method for facial expression recognition, automatic facial expression recognition has entered a new era.

With the development of expression researches, scholars focus on a more subtle expression research, that is, the micro expression research. It is a kind of short-lived facial expression made unconsciously by human beings when they try to hide some emotions. They correspond to seven universal emotions: disgusted, angry, fearful, sad, happy, surprised and contempt. The duration of micro expression is only 1/25 to 1/5 s, which expresses the real emotion that a person tries to suppress and hide. Although a subconscious expression may last only a moment, and sometimes it expresses the opposite emotion.

The main application fields of facial expression recognition technology include human–computer interaction, intelligent control, security, medical, communication, education, fatigue detection, political election and other fields. For distance education, teachers can judge the current learning situation of students by analyzing the expression status of students during class. For smart medical care, doctors can understand the degree of patients’ cure by capturing the expression on the patients’ faces. In short, with the progress of science and technology and the continuous development of psychology, the researches on facial expression will be more and more deep, the content will be more and more rich, and the application will be more and more extensive.

However, no matter the macro basic expressions or micro expressions, in the real interaction, a single expression can not fully express the complex emotional display of human beings. A major recent development in this issue is an article published on PNAS in 2014 [6]. This study proposes the concept of a compound expression and points out that multiple discrete basic expressions can be combined to form compound expressions. For example, when people encounter unexpected gifts, they should be happy and surprised. Therefore, in addition to the six common expressions of happy, surprised, sad, angry, disgusted and fearful, there are 21 distinguishable compound expressions, such as surprise and joy, sadness and anger. At present, most researchers focus on the researches of basic expression recognition, but few on compound expression recognition. The research of compound expression is a more powerful impetus for us to understand people’s inner feelings more accurately and truly. Therefore, in this paper, we pay our attention to the study on compound expression recognition.

In real life, we can recognize facial expressions by facial information. Generally speaking, facial expression features can be divided into texture and geometry based according to the expression content of facial images. In this work, we mainly start from those two aspects, proposing a scheme of combining spatial and frequency domains to implement end-to-end joint training based on model ensembling between models for appearance and geometric representations learning for the recognition of compound expressions in the wild. For appearance feature acquisition, we adopt the idea of transfer learning, introducing the ResNet50 model [7] pretrained on VGGFace2 [8] for face recognition to implement the fine-tuning process. Here, we try and compare two minds, one is that we utilize two static expression databases FER2013 [9] and RAF Basic [10,11] for basic emotion recognition to fine tune, the other is that we fine tune the model on the three channels composed of images generated by DWT2 and WAVEDEC2 wavelet transform based on rbio3.1 [12] and sym1 [13] wavelet bases respectively. For geometric feature acquisition, we firstly introduce the densesift [14] operator to extract facial key points and their histogram descriptions. After that, we introduce deep Stack AutoEncoder (deep SAE) [15], stacked Long Short Term Memory (LSTM) [16] and Sequence-to-Sequence with LSTM [17] and define their structures by ourselves. Then, we feed the key points and their descriptions into the input three models to train respectively and compare their performances. When the model training for appearance and geometric features learning is completed, we combine the two models with category labels to achieve further end-to-end joint training considering that ensembling the models which describe different information can further improve recognition results. Finally, we validate the performance of our proposed framework on the RAF Compound database.

The rest of this paper is arranged as follows: Section 2 discusses the recent development on facial expression recognition and gives an explanation to our proposed framework; Section 3 reports the experiments and results; Section 4 and Section 5 concludes our proposed framework and proposes the challenges on compound expression.

## 2. Materials and Methods

### 2.1. Related Work

The generation of facial expressions is a very complicated process. If psychological and environmental factors are not taken into account, what is presented to the observers is the simple muscle movements, and the resulting changes in facial shape and appearance. The static image presents the expression state of a single image when the expression occurs, and the dynamic sequence presents the motion of the expression between multiple images. Considering that the researches on compound expression recognition are still relatively few, and the related databases are relatively lacking, it is very necessary for better human–computer interaction. Therefore, in this paper we are aimed at the exploration of static compound expression recognition under the natural scenes.

After years of development, automatic recognition of facial expressions has been formed into a complete system. It mainly includes three key steps, namely face preprocessing, expression feature extraction, and classification. Face preprocessing usually includes face detection and alignment, illumination normalization, geometric pose normalization. With the gradual maturity of face recognition technology, many algorithms for face detection and alignment have been developed. For example, Viola-Jones [18] proposed to use a Haar operator to extract facial features and Adaboost to judge the faces. It can detect frontal faces quickly and accurately. As a large number of studies continue, researchers have found that detecting facial landmarks can be more conducive to the extraction of salient features. Therefore, statistical modeling is performed using shapes and textures, and the two statistical models of shape and texture are further fused into an appearance model which is called Active Appearance Model (AAM) [19]. Mixtures of Trees (MOT) [20] and Discriminative Response Map Fitting (DRMF) [21] express facial information by describing changes of local textures. Furthermore, cascading through regression models, there are many stacked models that are dug, such as supervised descent method (SDM) [22], Cascaded CNN [23], Tasks-Constrained Deep Convolutional Network (TCDCN) [24] and Multi-task CNN (MTCNN) [25] for multi-target detection and alignment.

For illumination normalization, under different lighting conditions, different people present the same expression or the same person presents different expressions, the intensity and contrast of the illumination will show great differences. However, these differences will affect our classification of an expression more or less. Thus, some illumination normalization algorithms have been introduced and promoted, such as Isotropic Diffusion (IS) [26], Discrete Cosine Transform (DCT) [27], Difference of Gaussian (DoG), Hormophobic filter [28] and Histogram Equalization (HE) [29,30] argued that assigning different weights on the Histogram Equalization and linear mapping features and fusing them linearly can overcome the drawbacks in HE. Furthermore, some prominent algorithms for geometric pose normalization are discussed in [31,32,33].

When it comes to feature extraction, currently, according to the degree of characterization information, methods for extracting appearance and geometric features of expressions can be classified into traditional feature engineering and deep learning based methods. Many handcrafted operators are utilized to present shallow representations. Local Gradient Coding (LGC) [34], Local Directional Pattern (LDP) [35] and LBP [36] are introduced to mine the pattern of texture changes in expression samples. Scale Invariant Feature Transformation (SIFT) [14] is argued to extract key points and histogram information based on key points which is robust to scale, rotation and translation. Three-dimensional information including time series can be obtained by LBP-TOP [37], HOG-TOP [38] and Pyramid Histogram of Oriented Gradients (PHOG-TOP) [39]. Non-negative matrix factorization (NMF) [40], Principle Component Analysis (PCA) [41] and sparse representation [42] are considered to save memory space and present significant information expression.

In recent years, the scale of expression databases has been expanded, showing rich diversity, and the computer hardware processing ability has been greatly improved, as well as the successful application of deep learning technology in various fields. Facial expression recognition technology is gradually moving from laboratory based tests to challenging real scene recognition. The ability of traditional handcrafted descriptors to express information is limited, most of which are shallow feature representations, failing to capture changes unrelated to expressions. Due to the powerful learning ability of deep learning technology, deep learning technology is gradually becoming widely used in facial expression recognition. Deep Belief Network (DBN) [43] is used to learn feature representations in a semi-supervised way. Deep AutoEncoder (DAE) [15] and Deep Sparse AutoEncoder (DSAE) [44] models code samples in an unsupervised way, learning a small number of features with great contribution to classify the expressions. The Convolutional Neural Network (CNN) [45] model is introduced because of its robustness to affine transformations. Based on the improvement of the CNN model, Region-CNN (R-CNN) is proposed in [46]. For dynamic sequences, 3D CNN [47] is utilized to present the spatial-temporal information between continuous frames, and the Recurrent Neural Network (RNN) [48] is a another structure that can express time series information well. Long Short Term Memory (LSTM) [16], which is proposed by Hochreiter and Schmidhuber, is a representative RNN. It has been widely promoted for dynamic expression recognition. Goodfellow et al. [49] introduced the Generative Adversarial Network (GAN), which is mainly for generating samples by training against each other through generator and discriminator. The Conditional Generative Adversarial Network (cGAN) [50] is argued to generate neutral faces corresponding to expression samples. Then expressive information can be captured in the cGAN model [51].

For the classification phase, traditional classifiers such as Support Vector Machine (SVM) [52], Adaboost [53], random forest [54], decision tree [55], K-Nearest Neighbors (KNN) [56], naïve Bayes [57] are used to judge the expression categories. For deep learning, we can implement end-to-end learning. Furthermore, now some dynamic expression databases provide audio information, which means that in addition to visual information, we can also combine audio information to achieve multi-modal expression recognition. Multiple combining methods for visual and audio representations are explored in [58].

In this paper, we put our emphasis on the compound expression recognition in the wild, and investigate the performance of our recommended framework on the RAF Compound database.

### 2.2. The Proposed Method

**Figure 1:**
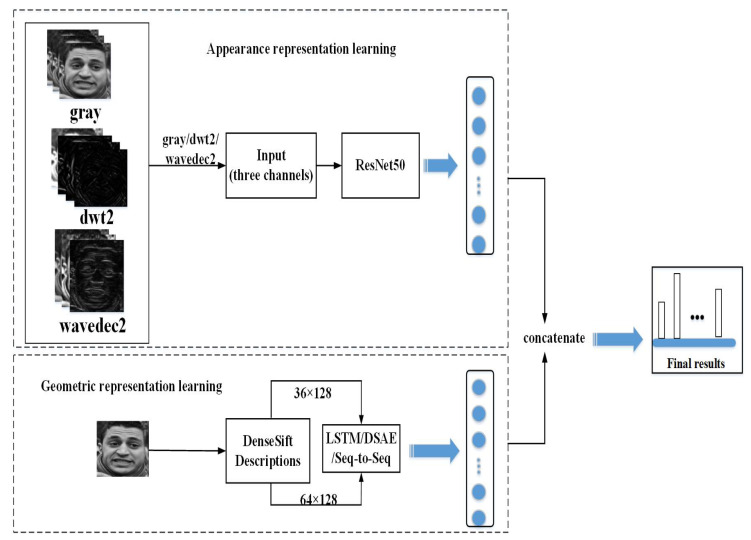
The diagram for our recommended scheme. Copyright reference: http://www.whdeng.cn/raf/model1.html#dataset.

In our work, we propose a scheme of combining spatial and frequency domain transforms to implement end-to-end joint training based on model ensembling between models for appearance and geometric representations learning for the recognition on compound expressions in the wild. The diagram for our recommended scheme is displayed in Figure 1. In the following sections, we will elaborate on the overall process in detail.

#### 2.2.1. Preprocessing Static Expression Databases

**Figure 2:**
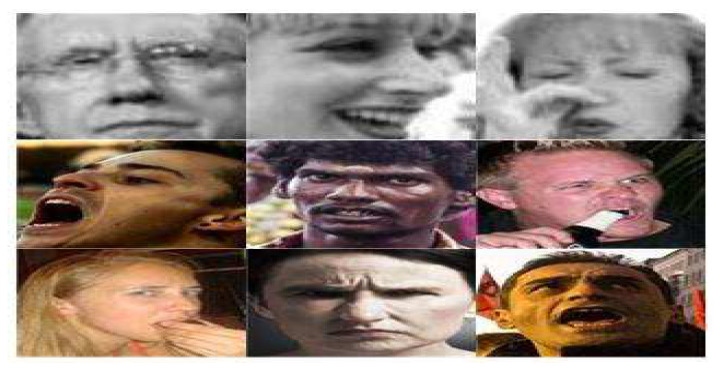
The samples of face detection and alignment based on Multi-task Convolutional Neural Network (MTCNN) in FER2013, Real-world Affective Faces (RAF) Basic and RAF Compound. The upper row presents samples in FER2013; the middle row presents samples in RAF Basic; the lower row presents samples in RAF Compound. Copyright reference for FER2013: https://www.kaggle.com/c/challenges-in-representation-learning-facial-expression-recognition-challenge/data; copyright reference for RAF database: http://www.whdeng.cn/raf/model1.html#dataset.

In our study, FER2013, RAF Basic and RAF Compound are introduced respectively. Among them, FER2013 and RAF basic are used to fine tune the model, and RAF Compound is used to evaluate our proposed framework. There will be many changes that have nothing to do with facial expression under natural scenes, such as different backgrounds, lighting conditions, head postures and so on. Therefore, before feature learning, we need to use preprocessing to calibrate and align the visual semantic information of the faces, and then face detection and alignment, illumination normalization are performed. Firstly, a Multi-task Cascaded Convolutional Network (MTCNN) is utilized to implement face detection and alignment for three static expression databases. MTCNN algorithm is a face detection and alignment method based on deep learning. It can accomplish the task of face detection and alignment at the same time. Compared with traditional algorithms, it has better performance and faster detection speed. Figure 2 shows the samples of face detection and alignment based on MTCNN in FER2013, RAF Basic and RAF Compound.

What we need to explain here is that we delete the samples with a large area of occlusion and no faces detected considering that such noise samples are not conducive to model learning.

Considering that uneven illumination will lead to poor recognition results, DOG filter, Contrast Limited Adaptive Histogram Equalization (CLAHE), and linear sharpening algorithms are introduced. Dog filter is mainly used to convolve the target images with a Gaussian function. This process is called denoising. CLAHE [59] is an improvement to traditional Adaptive Histogram Equalization (AHE). For an image, the contrast of different regions may vary greatly. Maybe some locations are bright and some locations are dim, it is not the best choice to use a single histogram to adjust. Therefore, based on the idea of block processing, an adaptive histogram equalization algorithm is proposed. However, sometimes this method will amplify some noise. Thus, CLAHE is proposed to overcome the drawbacks. Linear sharpening can make the edge, outline and details of the image clear.

Preprocessing can remove the noise and improve the quality of images, which is beneficial to the learning of the model.

#### 2.2.2. Frequency Domain Transform on Expression Samples

The frequency domain transform of the images mainly describes the intensity of the gray changes in the images. The high and low frequency of the images are measures of the intensity changes between the various positions of the images. The low frequency component is mainly a comprehensive measure of the intensity of the entire image. The high frequency component is mainly a measure of the edge and contour of the image.

The signal tends to be simpler and more intuitive in the frequency domain than in the time domain and the wavelet transform is a major breakthrough for Fourier transform. It overcomes the defect that Fourier transform cannot handle some local signals very well. The change of wavelet is to replace the infinitely long trigonometric base in the Fourier transform with a finite-length attenuated wavelet base. Thus, it has excellent time-frequency domain characteristics and can effectively analyze local stationary signals. At the same time, it has good energy concentration characteristics, which can be encoded in the transform domain, and obtain higher compression efficiency. Formula (1) presents the wavelet transform.

(1) $$WT\left(a,\tau \right)=\frac{1}{\sqrt{a}}\int_{-\infty }^{\infty }f\left(t\right)*\psi \left(\frac{t-\tau }{a}\right)dt$$

**Figure 3:**
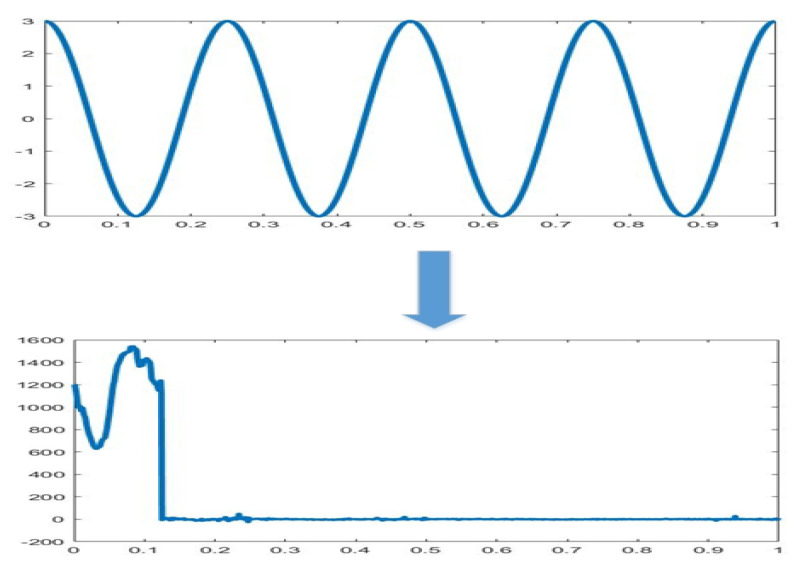
The effect of the wavelet transform compared with the Fourier transform. The upper denotes the effect of Fourier transform; the lower denotes the effect of the wavelet transform.

It can be seen from the formula that unlike the Fourier transform where the variable has only the frequency ω, and the wavelet transform has two variables: the scale a (scale) and the translation t (translation). The scale a controls the expansion and contraction of the wavelet function, and the translation amount t controls the translation of the wavelet function. The scale corresponds to the frequency (inverse ratio), and the amount of translation t corresponds to time. Compared with the Fourier transform, the effect of the wavelet transform is shown in Figure 3.

In our research, we employ 2D Discrete Wavelet Transform (DWT2) and WAVEDEC2 to realize the frequency domain transform. DWT2 is a two-dimensional single-scale discrete wavelet transform. It can perform two-dimensional single-scale wavelet decomposition by specifying wavelet or decomposition filter. WAVEDEC2 is a two-dimensional multi-scale wavelet decomposition. Sym1 and rbio3.1 are used as the base functions of the above two wavelet transforms. Sym, to some extent, can reduce the phase distortion of signal analysis and reconstruction; rbio can generate reconstruction and decomposition scaling filters. The ‘rbio3.1’ wavelet has three vanishing moments for the decomposition wavelet and one vanishing moment for the reconstruction wavelet. For WAVEDEC2 based on sym1 and rbio3.1, a two-layer transformation is performed and extracts the results of the first layer.

**Figure 4:**
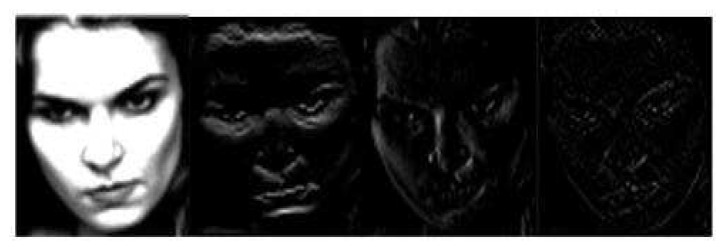
The illumination normalized expression samples of frequency domain transform based on DWT2 in RAF Compound. Approximate component, horizontal detail component, vertical detail component, diagonal detail component from left to right. Copyright reference: http://www.whdeng.cn/raf/model1.html#dataset.

**Figure 5:**
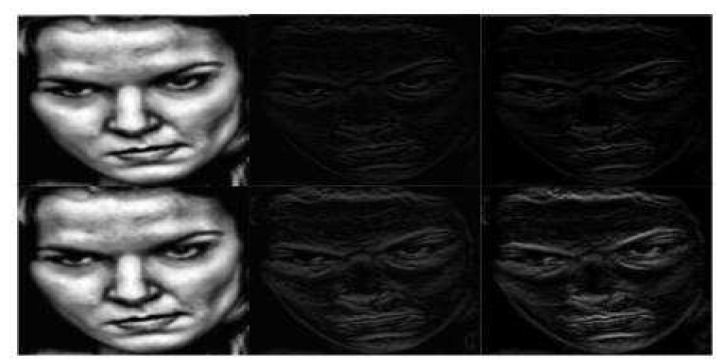
The illumination normalized expression samples of frequency domain transform based on WAVEDEC2 in RAF Compound. The upper row denotes illumination normalized expression samples of WAVEDEC2 transform based on sym1; the lower row denotes illumination normalized expression samples of WAVEDEC2 transform based on rbio3.1. Copyright reference: http://www.whdeng.cn/raf/model1.html#dataset.

We generate the frequency domain images of FER2013, RAF basic and RAF compound based on the above two different wavelet transforms and wavelet bases respectively. For DWT2, each introduced wavelet base produces four outputs, representing approximate component, horizontal detail component, vertical detail component and diagonal detail component respectively. For WAVEDEC2, each introduced wavelet base produces two outputs, denoting low frequency and high frequency components respectively. DoG filter is firstly performed on low frequency images to achieve initial illumination normalization, then CLAHE is utilized to further adjust the local illumination of the images which have been processed by DoG filter. CLAHE and linear sharpening are also explored on high frequency images separately. We want to compare smoothing and sharpening which can make the information carried by high frequency images more prominent and beneficial to extract salient features. The illumination normalized expression samples of frequency domain transform based on DWT2 and WAVEDEC2 in RAF Compound are presented in Figure 4 and Figure 5 separately.

#### 2.2.3. Appearance Feature Acquisition Based on Transfer Learning for Resnet50 with Samples in Spatial and Frequency Domain

In our recommended scheme, we mainly study the appearance and geometric representations of the compound expressions. Due to the small number of expression samples, too shallow of a model is not conducive to the capture of abstract information. Because deep learning has strong migration ability in some similar scenarios and we want to get better performance in deep learning models with small databases, the mind of transfer learning is introduced. Transfer learning can be understood as applying mature knowledge in one field to other scenarios. If the two scenarios are very similar, the model for transfer learning can achieve satisfactory results with a slight change in structure and a small amount of training and vice versa.

Face technology is the foundation of expression technology, and with the rapid development of face detection and recognition technology, expression recognition technology has been gradually improved. Therefore, for faster training and more accurate recognition results, ResNet50 model pretrained on the VGGFace2 database for face recognition is utilized to fine tune given that expression describes the movement and transformation of facial muscles, making some shallow features of the face and expression have commonality.

**Figure 6:**
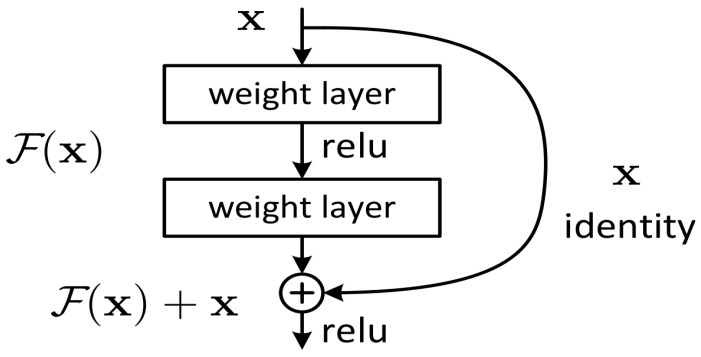
The key structure of ResNet.

With the deepening of the network, the accuracy of the training set decreases. We can be sure that this is not caused by overfitting. So a new network called deep residual network is proposed to solve the problem of performance degradation after the network deepens. It proposes two kinds of mapping: one is identity mapping and the other is residual mapping, so the final output is y=F(x)+x. The key structure of ResNet is depicted in Figure 6.

The appearance feature describes the texture distribution pattern of the images and vividly presents the image content. For the mining of appearance features, we have made two attempts, one is based on gray-scale images, the other is based on frequency-domain transform images. For the learning in the space-time domain, gray images in FER2013 and RAF Basic are used to fine tune ResNet50 respectively, because FER2013 and RAF basic contain seven kinds of basic expressions which are the basis of forming compound expressions. After fine tuning, we started to train the model on the RAF Compound’s training set and test it on its test set.

For the learning in the frequency domain, we fine tune ResNet50 using images based on frequency domain transform for FER2013 and RAF Basic. Before fine tuning the model, we first generate the frequency domain images based on DWT2 and WAVEDEC2. For outputs of DWT2 which are expressed as Formula (2), we set up two groups, one is to take the approximate component (CA), the horizontal detail component (CH) and the vertical detail component (CV) as the three channels of input samples, the other is to take the horizontal detail component (CH), the vertical detail (CV) component and the diagonal component (CD) as the three channels of input samples for model training. For outputs of WAVEDEC2 transform based on rbio3.1 and sym1 wavelet bases, which can be presented in Formulas (3) and (4), we use the original gray images, low frequency component (C) and high frequency component (S) as three channels of input samples for model learning. When the fine tuning of the model is completed, the training based on the samples of frequency domain transform in the RAF Compound is started.

(2) $$\left[\mathrm{CA},\mathrm{CH},\mathrm{CV},\mathrm{CD}\right]=\mathrm{dw}t2\left(X,‘\mathrm{rbio}3.1’\right)$$

(3) $$\left[C,S\right]=\;\mathrm{wavedec}2\;(X,2,\;‘\mathrm{rbio}3.1’)$$

(4) $$\left[C,S\right]=\;\mathrm{wavedec}2\;(X,2,\;‘\mathrm{sym}1’)$$

### 2.3. Deep Learning on Densesift Descriptions for the Extraction of Geometric Features

The geometric features of an image refer to the features of the object’s position, orientation, perimeter, and area in the image. Although the geometric features of the image are relatively straightforward and simple, they play an important role in many image analysis problems. Here, we mainly train three different deep models on densesift descriptions for mining geometric information on expression samples.

#### 2.3.1. Descriptions for Densesift Operator

For data selection to generate geometric features, the densesift operator is used to extract the salient feature points of the face and histogram descriptions of the feature points. The densesift algorithm is a feature extraction process that performs block processing on the input images and performs SIFT operation on each block. Densesift can properly meet the feature representation ability of images under different classification tasks according to the adjustable parameters’ size.

Densesift is often the first step in feature extraction in non-deep learning models. When the sampled points are extracted from the SIFT descriptor, after the codebook projection, the sample points are projected on the same codeword, which represents a set of similar descriptors. The ability to distinguish between sample points is different between different codewords (equivalent to each bin of the histogram). Densesift does not attempt to use a classifier to determine whether it is a salient point when looking for a salient point, but for simplification, all significant points are equally dense across the various regions of the images.

#### 2.3.2. Descriptions of Each Deep Learning Model Used to Extract Abstract Geometric Features

For model selection to train on densesift descriptions, we have separately defined the model structures of SAE, LSTM, Sequence-to-Sequence to train. We introduce some time series models under such assumption that when an expression occurs, each key point extracted is changed according to a certain timing.

**Figure 7:**
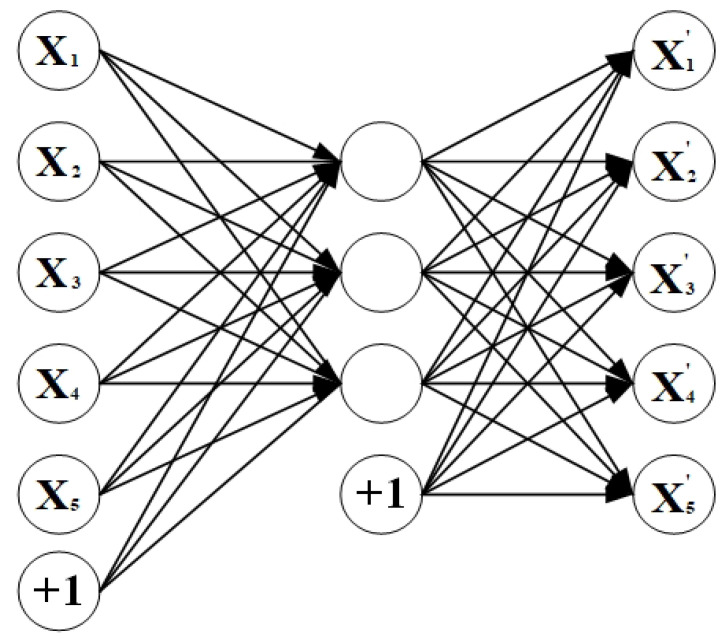
The main body of the simple AutoEncoder (AE).

AutoEncoder (AE) is an unsupervised learning technique that uses neural networks for feature representation. That is, we design a neural network architecture that imposes a “bottleneck” in the network, forcing the original input to compress the knowledge representation. This compression and subsequent reconstruction will be a very difficult task if the input features are independent of each other. However, if there are some structures in the data (i.e., there is a correlation between the input features), then those structures can be learned and used when forcing a bottleneck through the network. Sparse AutoEncoder actually adds sparse constraints to model so as to extract the main features and achieve the effect of reducing dimensions. Furthermore, it also has some anti-noise ability and interpretability due to its sparsity. We can generate a deep SAE by stacking multiple AE structures. The main body of the simple AE is shown in Figure 7.

In the traditional RNN, BackPropagation through Time (BPTT) [60] is used in the training algorithm. When the time is long, the residuals that need to be returned will decline exponentially, resulting in the slow updating of network weights, which can not reflect the long-term memory effect of RNN. Therefore, a storage unit is needed to store the memory, namely, the LSTM. LSTM is a special RNN model, which added a method of carrying information across multiple time steps to solve the problem of gradient dispersion of the RNN model. Bidirectional LSTM is a variant of the normal LSTM, and it takes advantage of the sequential sensitivity of the RNN, consisting of two RNNs, each of which processes the input sequence in one direction (time positive order and time reverse order) and then merges their representations together in both directions. By processing the sequence in both directions, the bidirectional RNN is able to capture patterns that may be ignored by the one-way RNN.

**Figure 8:**
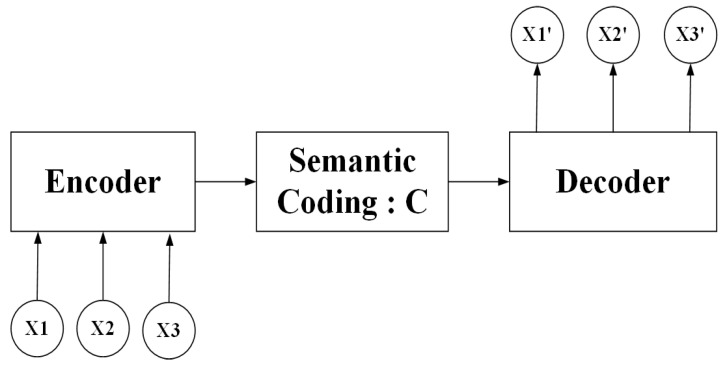
The algorithm framework in Sequence-to-Sequence.

The Sequence to Sequence [61] model was first developed by a Google engineer in 2014. The model is a kind of end-to-end algorithm framework, which is a transform model from sequence to sequence, applied in machine translation, automatic response and other scenarios. It is generally implemented by the encoder-decoder framework. The encoder converts a variable-length signal sequence into a fixed-length vector representation, and the decoder turns this fixed-length vector into a signal sequence of variable-length targets. The encoder and decoder sections can be any text, voice, image, or video data. The model can use CNN, RNN, LSTM, Gated Recurrent Unit (GRU), BLSTM, and so on. Figure 8 presents the algorithm framework in Sequence-to-Sequence.

**Figure 9:**
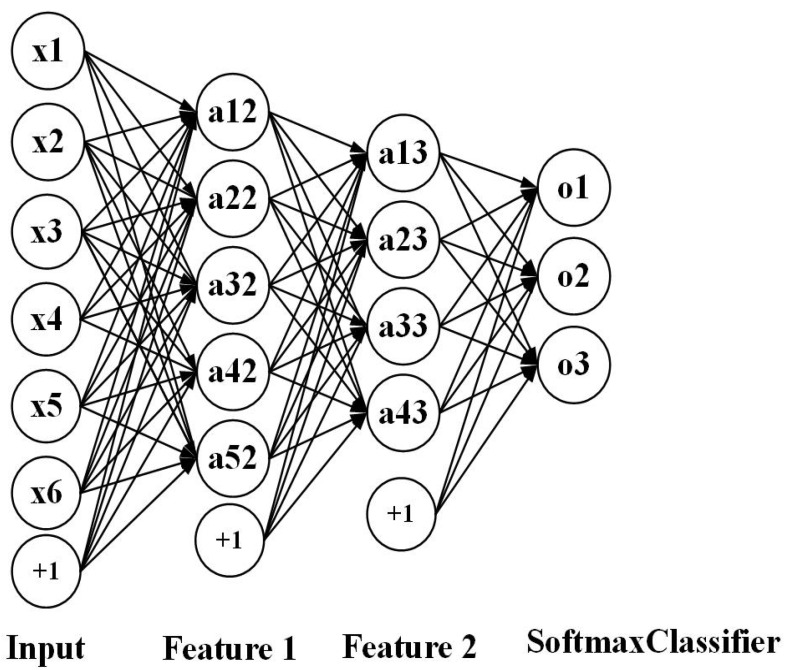
The encoder of deep Stack AutoEncoder (SAE) with softmax classifier.

In our work, we try to train deep SAE, stacked LSTM, stacked BLSTM and Sequence-to-Sequence model with stacked LSTM and stacked BLSTM based on densesift descriptions and compare their performances. In addition, it is worth noting that after the unsupervised training on deep SAE. We combine the encoder of deep SAE with the softmax classifier to further supervise training, which is displayed in Figure 9.

## 3. Results and Discussion

### 3.1. Introduction for FER2013, Raf Basic and Raf Compound

FER2013: the FER2013 facial expression database consists of 35,886 facial expression images, including 28,708 training pictures, 3589 public test pictures (PublicTest) and private test pictures (PrivateTest). Each gray image is fixed by size 48×48, there are seven kinds of expressions, corresponding to the digital label 0-6, the specific expression corresponding to the label is as follows: 0 angry; 1 disgusted; 2 fearful; 3 happy; 4 sad; 5 surprised; 6 neutral. Since FER2013 is only used for pretraining of models, in order to involve as many samples as possible in training, we merge the train and the private test set as a large training set, and use the public test set as the validation set to fine tune or pretrain. The distribution of each category sample in FER2013 is described in Table 1. (download link: https://www.kaggle.com/c/challenges-in-representation-learning-facial-expression-recognition-challenge/data).

RAF: Real-world Affective Faces database (RAF) was built by downloading more than 30,000 images in batches on Flickr image social networks using expression-related keywords, and use crowdsourcing technology to annotate these images. Those annotation results and the annotators’ reliability are evaluated based on the expectation maximization algorithm, which further filters out those noise labels for more accurate annotation results. The entire database contains nearly 30,000 images with a seven-dimensional expression distribution. According to the seven-dimensional expression probability distribution vector corresponding to each picture, we divide the database into seven basic expressions and eleven types of compound expressions (download link: http://www.whdeng.cn/raf/model1.html#dataset).

There is a big difference between the facial expression naturally revealed in people’s daily life and the facial expression uniformly regulated in the laboratory environment. In real life, different people express their expressions in various ways. Each expression will contain many different forms due to the different identities of the characters, which undoubtedly challenges the expression recognition in the real face world. In our research, we mainly focus on the compound expression recognition in the wild. Table 2 and Table 3 shows distribution of various samples in RAF Basic and RAF Compound separately.

### 3.2. Experiment Setups

We implement our experiments on the 64-bit ubuntu18.04 system with the TITAN RTX 2080TI GPU and pytorch framework.

For data settings, before model training for appearance information mining, we resize the inputs into 224×224 pixels. Furthermore, we extract the densesift descriptions of 36×128 and 64×128, respectively, for geometric feature extraction considering that different numbers of key points may have different effects on the training of the model.

For model settings, we only replace the output layer of ResNet50 with the number of category tags for the current training set. In addition, we define the structures of deep SAE, stacked LSTM, BLSTM and Sequence-to-Sequence with stacked LSTM and BLSTM from scratch. We stack several LSTMs and a softmax layer to generate a stacked LSTM model, and we set the number of categories of the current training set to the number of nodes in the softmax layer. The stacked BLSTM model is built similar to the stacked LSTM model. In addition, the stacked LSTM and BLSTM are also embedded in the Sequence-to-Sequence model, which is concentrated on Sequence-to-Sequence mapping to generate compressed coding representations. The encoder and decoder for the deep SAE are built with several dense layers respectively. It is worth mentioning that the input for stacked LSTM and BLSTM and Sequence-to-Sequence is a three-dimensional vector, which contains batchsize, timesteps and input_features. However, the deep SAE’s input is a one-dimensional vector.

### 3.3. Experimental Results

We implement two training mechanisms for the extraction of appearance representations. One is based on gray images, the other is based on the input of the three channels composed of images of the frequency domain. There are four forms of frequency domain images based on different wavelet bases and illumination normalization methods: dwt2_firstthree (rbio3.1), which denotes CA, CH, CV of DWT2 transform based on wavelet basis rbio3.1; dwt2_latterthree (rbio3.1), which represents CH, CV, CD of DWT2 transform based on wavelet basis rbio3.1; wavedec2_rbiosharpen, which describes WAVEDEC2 transform based on wavelet basis rbio3.1 and the linear sharpening method is employed on a high frequency component; and wavedec2_symsharpen, which depicts WAVEDEC2 transform based on wavelet basis sym1 and the linear sharpening method is employed on a high frequency component, respectively. For the training process, we first fine tune the ResNet50 model on FER2013, then the RAF Basic is utilized to further train the fine-tuned model based on FER2013 for two learning mechanisms. Finally, three-fold cross-validation and grid search is introduced to utilize RAF Compound to train the ResNet50, which is fine tuned on FER2013 and RAF Basic.

The model parameters for appearance feature learning are set as follows: we set the epoch to 20; 0.001, 0.0001, and 0.00001 are selected to find the best learning rate, the size of batch is set to 64 or 128; and Adam, RMSprop, SGD are tried separately. It should be noted that fine tuning ResNet50 on dwt2_latterthree (rbio3.1) performs worse than other three frequency domain transforms, thus, dwt2_latterthree (rbio3.1) was not considered to continue subsequent learning on RAF Compound. The results of the RAF Compound test set based on different input channels of ResNet50 fine tuned on FER2013 are shown in Table 4; the results of the RAF Compound test set based on ResNet 50 pretrained on FER2013 and RAF Basic are displayed in Table 5.

By comparing Table 4 and Table 5, we find that the model just fine tuned on FER2013 performs worse on the test set of RAF Compound than the model fine tuned on FER2013 and RAF Basic although there are some slight differences in data presentation between the two databases. It can be concluded that databases with similar contents can be beneficial to train the model and achieve good results in the final task. Table 4 and Table 5 also confirm that for small databases that want to achieve better results on deeper and more complex models, transfer learning is a very good choice.

In addition, in our research, we fine-tune the ResNet50 with spatial and several different forms of frequency-domain images, respectively. We argue that the spatial and frequency domains provide us with different perspectives. The shape of the signal can be directly observed in the space-time domain, but the signal cannot be accurately described with limited parameters. However, in the frequency domain, some features are more prominent and easy to process. For example, it is difficult to find the noise pattern in the spatial images. If it is transformed into the frequency domain, it is better to find the noise pattern and it can be more easily processed.

**Figure 10:**
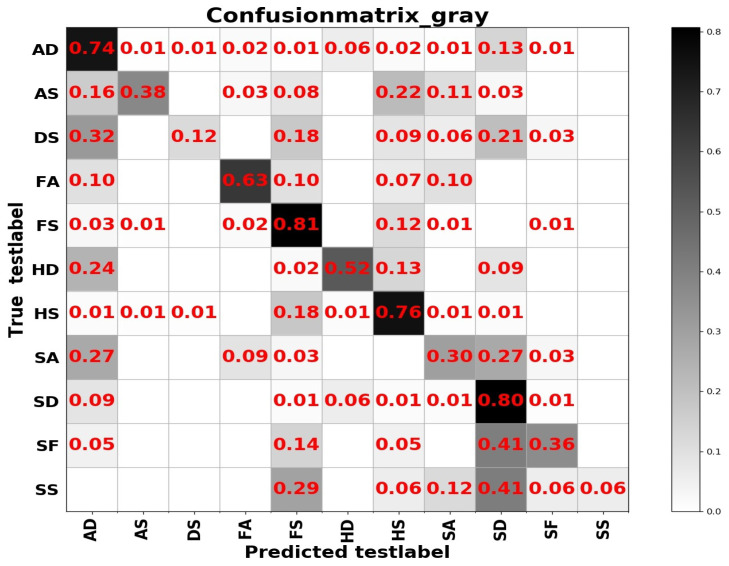
The confusion matrix of the RAF Compound test based on the input channels composed of spatial images.

**Figure 11:**
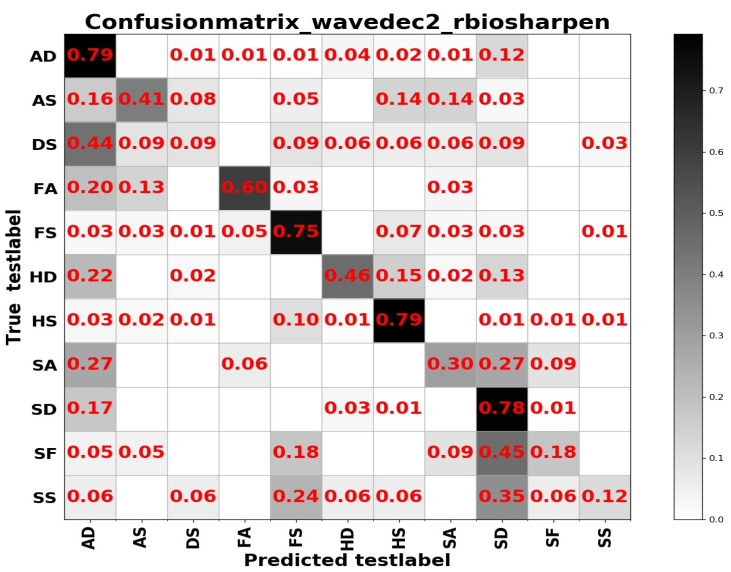
The confusion matrix of the RAF Compound test based on the input channels composed of the combination of spatial and frequency domain images under wavelet base rbio3.1.

**Figure 12:**
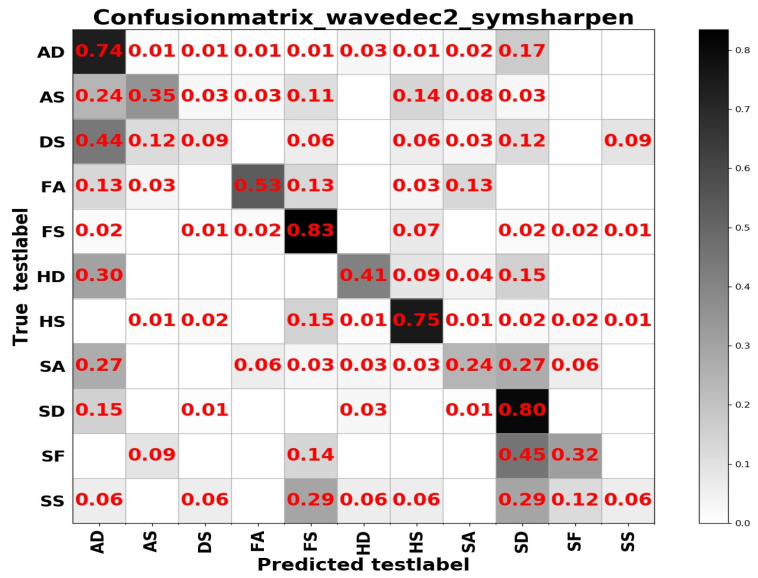
The confusion matrix of the RAF Compound test based on the input channels composed of the combination of spatial and frequency domain images under wavelet base sym1.

**Figure 13:**
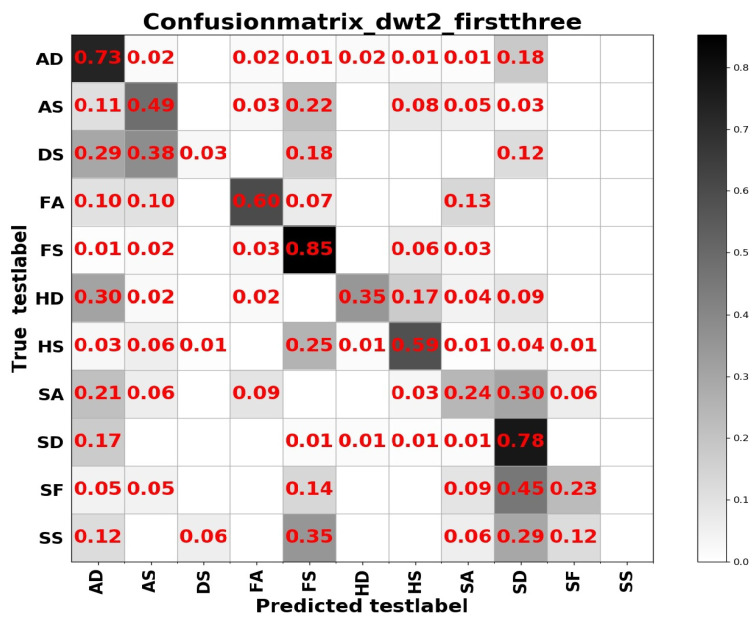
The confusion matrix of the RAF Compound test based on frequency domain images under rbio.3.1.

From Table 4 we can see that test accuracy on the input data composed of a combination of frequency and spatial domain images is the best, while the model trained on spatial images achieves slightly higher results in Table 5. Investigating the reason, we think that the difference between the expressions of the two sample sets has a lot to do with it. In summary, from the results of the two tables above, the learning based entirely on the frequency domain is the worst, while the learning combining the spatial and frequency domains shows more outstanding performance. The confusion matrices on the RAF Compound test set based on different input channels for ResNet50 pretrained on two databases for the recognition of basic emotions are presented in Figure 10, Figure 11, Figure 12 and Figure 13.

From the confusion matrices, we can derive that imbalance of various samples leads to huge differences in recognition results. The imbalance of various types of samples leads to huge differences in recognition results. The recognition accuracy of categories with a larger number of samples is relatively high, and vice versa. Some expressions with similar facial muscle movements are easy to misrecognize. For example, it is easy to mistake ‘sadly fearful’ as ‘sadly disgusted’, especially for complex expressions, which are composed of multiple emotions, it is easier to be misclassified.

In addition to extracting the appearance information to express the texture features, we also extract the key points of the faces and the histogram descriptions based on the key points to achieve the exploration of geometric features. In this regard, we have introduced a total of two types of models, one is an unsupervised AutoEncoder and the other is a sequence model. We defined a stacked AutoeEncoder from scratch and two sequence models which includes LSTM and Sequence-to-Sequence.

Same as the pretraining strategy for models used for appearance representations, we pretrain our model continuously on FER2013 and RAF Basic for unsupervised and supervised learning. Based on being able to capture more distinctive features of each image by encoding itself, AutoEncoder is considered given that the occurrence of expressions, the process of performing actions at significant key points on the face also occurs in a certain order, and different expressions have different orders, sequence models are selected. Table 6 presents the performance of sequence models and AutoEncoder on the RAF Compound test set.

From Table 6, we can see that DSAE+Softmax achieves the best results compared to other models for geometric representation learning. Introducing AutoEncoder to reconstruct the samples in an unsupervised way and by minimizing the reconstruction error, the model structures and parameters are optimized to extract the most expressive information for the samples firstly. Then, we combine the encoder with the softmax layer and incorporate the category labels to realize supervised learning. Based on that, we can also infer that applying unsupervised and supervised learning to some recognition tasks may achieve better performance than a single learning method.

Furthermore, from Table 6, we also notice that Sequence-to-Sequence with LSTM or BLSTM is also a self-encoding unsupervised learning mode, which is just a sequence-to-sequence self-encoding mode. In our work, we use its implementation principles to carry out sequence-to-sequence modeling on a series of extracted facial key points and their histogram descriptions. In order to avoid underfitting caused by insufficient sample sizes, we extracted densesift descriptions of two sizes respectively, but the performance is so unsatisfactory. The possible causes of that result are, as a sequence expression, its context information is not closely connected, time information is also not obvious, and the feature size and number of samples are not enough by observing that inputs with 64×128 densesift descriptions outperform 36×128.

In addition, for the simply supervised training mode, BLSTM is better than LSTM based on the results presented in Table 6. We can conclude that in the case of avoiding overfitting, bidirectional sequence learning is helpful for mining more useful information.

After completing the model training for appearance and geometric information, model ensembling technology is utilized to integrate appearance and geometric representations to generate more comprehensive information and achieve better recognition results. The accuracy rates of the RAF Compound test set based on different model ensembling strategies are shown in Table 7 and Table 8.

From Table 7 and Table 8, it can be observed that model ensembling can effectively improve the recognition effect by observing that most ensembling strategies achieve better results on the final task compared to the previous single model. And the combination of unsupervised and supervised learning is still far ahead of other models based on the recognition results generating from the model ensembling between ResNet50 and DSAE+Softmax. Furthermore, the performance of model ensembling between ResNet50 and Sequence-to-Sequence on spatial and frequency domain images is better than spatial images. It can also be inferred that the sequence model is more suitable for frequency domain images that are more sensitive to timing signals.

An interesting discovery is that model ensembling between ResNet50 trained on spatial and frequency domain images and Seq-to-Seq with LSTM or BLSTM+Softmax achieves the satisfactory recognition rates in Table 7. Based on that, we can infer that model diversity plays a vital role in model ensembling and it is not how good your best model is.

## 4. Discussion

**Figure 14:**
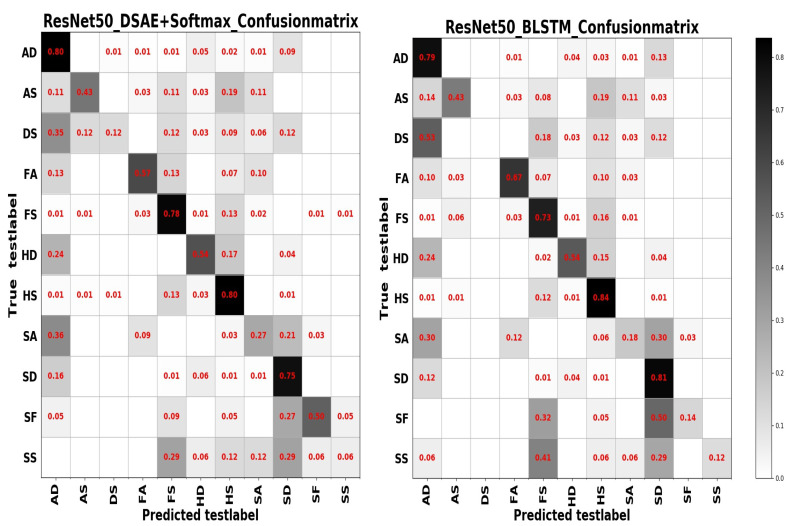
The confusion matrix on the RAF Compound test set for the model ensembling of ResNet50 and DSAE+Softmax (**left**); the confusion matrix on the RAF Compound test set for the model ensembling of ResNet50 and BLSTM (**right**).

**Figure 15:**
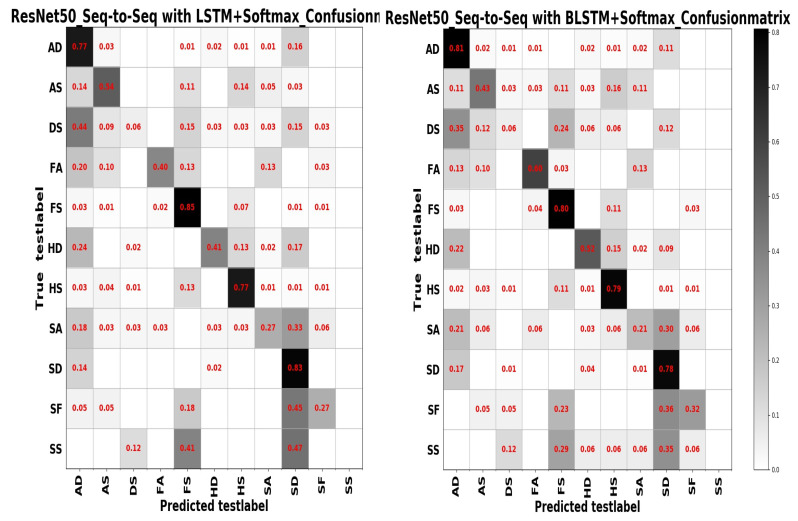
The confusion matrix on the RAF Compound test set for the model ensembling of ResNet50 and Seq-to-Seq with LSTM+Softmax (**left**); the confusion matrix on the RAF Compound test set for the model ensembling of ResNet50 and Seq-to-Seq with BLSTM+Softmax (**right**).

The confusion matrices based on different model ensembling strategies are displayed in Figure 14 and Figure 15. Based on different model ensembling strategies, the learned information is also different, and from the confusion matrices, we can observe that for categories with a small number of samples, the recognition results are different for different model ensembling strategies, some can get a small recognition rate, some can get a high recognition rate, but some results are 0. This also precisely shows that although we have introduced the model ensembling technology, we have only ensembled two models, and the diversity of models is insufficient, resulting in the learned information not being comprehensive. There are significant shortcomings in both the number of models and the diversity of models.

All in all, for our final task, the ensembling of different models is still very beneficial. We think the framework we propose is flexible and extensive. First of all, in terms of the form of features, we combine texture and geometric features, which will make the expression of features more comprehensive; second, in terms of the basic properties of the signal, we combine the temporal and spatial images, which allows us to analyze the signal changes in the images from different angles; third, in terms of the model, we introduce the convolutional neural networks to describe the spatial information and the recurrent neural networks to describe the temporal information respectively; fourth, as for the inputs of the model, we consider both the information of the whole images and the information of the local image blocks. As for the learning of geometric features, we define the model structures ourselves, so other researchers can redefine the model related to their tasks according to their own needs, which is more flexible. In addition, the model used for texture feature learning can also be replaced with other models. Based on the combination of the two, when implementing end-to-end training, the training is relatively fast. We believe that the framework we recommend has a certain reference for application in real life.

The abbreviations of the above expression categories are shown in Table 9.

However, due to the lack of researches on compound expressions, we cannot directly compare with other researchers’ works on this topic. Therefore, we have taken several traditional classic operators that describe texture and geometric information on our database to extract features and a SVM classifier is introduced to recognize. In addition, we employ four-fold cross-validation and grid search methods to train and select the best parameters for SVM. Results of the comparison are listed in Table 10. From Table 10, we found that our proposed combination of texture and geometric representation based on traditional and deep learning methods performs better than the single traditional feature engineering methods.

## 5. Conclusions

In this study, we propose a framework of combining spatial and frequency domain transform to implement end-to-end joint training based on model ensembling between models for appearance and geometric representations learning for the recognition of compound expressions in the wild. In our scheme, we explore the appearance features based on the spatial and frequency domains respectively. In addition, we dig the more abstract geometric information based on training the models of different structures with densesift descriptions, which are extracted from gray images. When the two models complete the training independently, we conduct model ensembling based on independent models with large structural differences for further training to get more comprehensive information and more accurate results. Finally, the validity of our recommended framework is verified on RAF Compound. At the same time, our work also shows that model learning based on the combination of spatial and frequency domains is better than single-form input. Furthermore, model ensembling is a very effective way to improve the final recognition results and there is much more room to mine. In addition, much more attention should be paid to the model diversity for the learning of complementary information.

Another point is that although there have been many studies on seven basic expressions, theresearches on the compound expressions are far from enough. There are some questions that we need to consider. So far, the defined categories of compound expression cover only a small part. Compound expressions are not just about two basic emotions, they may have more emotions happening at the same time. In addition, an old-fashioned question is one of unbalanced sample distribution across categories, leading to poor recognition performance on the categories with small size. Annotation of samples is also an arduous task due to annotators with different backgrounds, cultures and ages. Furthermore, the features learned only based on visual modality may not be complete. We need to introduce more modalities to achieve comprehensive and complementary feature mining.

In addition, in future work, we will make improvements in the following aspects: for the learning of texture features, in the final feature extraction, add a layer of feature transformation based on a nonlinear injective function, which retains more potential information. For the learning of geometric features, we introduce a recurrent network model that describes time series information based on local image blocks. For this part, considering that when facial expressions occur, facial actions occur in a certain time sequence, and the contribution of each local block is different. Therefore, the attention mechanism in NLP can be introduced into the recurrent neural network, such as self-attention and multi-head attention. What is more, the convolution models currently used are all based on the data with Euclidean structure to achieve feature learning. However, most data in real life exists in non-Euclidean structures, but the convolutional neural network models seem to be invalid in the face of such a data structure, because it has no translation invariance. In order to find a model suitable for this structure, graph neural network (GNN) came into being, and then graph convolutional neural network was also proposed. However, not all data have some kind of adjacency relationship, forming a topological graph, so that poses another challenge for the application of graph convolutional neural networks. In order to solve this problem, dynamic Graph Convolutional Network (GCN) was introduced, based on a certain similarity calculation method to dynamically find the K neighbors of each node, and then realize the aggregation of the features of the neighboring nodes. In terms of feature aggregation for GCN, whether the aggregation function has a nonlinear injective property plays a vital role in the performance of the entire model. We are considering combining the model based on data with Euclidean structure and the model based on the data with non-Euclidean structure to achieve end-to-end training, using the input of the former as the output of the latter for joint training. In addition, in the division of local image blocks, we can try to obtain local image blocks based on 68 facial landmarks as the center. Such local blocks may be more sensitive to changes in facial movements.

All in all, there are many more spaces to explore, especially based on compound expression recognition under natural scenes. 

## Figures and Tables

**Table 1:** The distribution of each category sample in FER2013.

Basic Expressions	Train + PrivateTest	PublicTest
Angry	4486	467
Disgust	490	56
Fear	4625	496
Happy	8094	895
Neutral	5591	607
Sad	5424	653
Surprise	3587	415

**Table 2:** Distribution of various samples in RAF Basic.

Basic Expressions	Train	Test
Angry	705	162
Disgust	717	160
Fear	281	74
Happy	4772	1185
Neutral	2524	680
Sad	1982	478
Surprise	1290	329

**Table 3:** Distribution of various samples in RAF Compound.

Compound Expressions	Train	Test
Angrily Disgusted	667	174
Angrily Surprised	138	38
Disgustedly Surprised	113	35
Fearfully Angry	117	33
Fearfully Surprised	444	116
Happily Disgusted	219	47
Happily Surprised	562	135
Sadly Angry	130	33
Sadly Disgusted	597	141
Sadly Fearful	107	22
Sadly Surprised	68	18

**Table 4:** The results of the RAF Compound test set based on different input channels of ResNet50 fine tuned on FER2013.

Model	InputChannels	TestAccuracy
ResNet50	Gray	57.12%
ResNet50	Gray+wavedec2_rbiosharpen	58.81%
ResNet50	Gray+wavedec2_symsharpen	58.94%
ResNet50	dwt2_firstthree(rbio3.1)	53.63%

**Table 5:** The results of the RAF Compound test set based on different input channels of ResNet 50 pretrained on FER2013 and RAF Basic.

Model	InputChannels	TestAccuracy
ResNet50	Gray	65.80%
ResNet50	Gray+wavedec2_rbiosharpen	65.41%
ResNet50	Gray+wavedec2_symsharpen	64.28%
ResNet50	dwt2_firstthree(rbio3.1)	60.88%

**Table 6:** The performance of sequence models and autoencoder on the RAF Compound test set.

Model	Inputs	TestAccuracy
LSTM	36×128 dsift descriptions	37.44%
	64×128 dsift descriptions	37.82%
BLSTM	36×128 dsift descriptions	41.32%
	64×128 dsift descriptions	37.56%
DSAE+Softmax	4608 dsift descriptions	41.45%
Seq-to-Seq with LSTM+Softmax	36×128 dsift descriptions	33.81%
	64×128 dsift descriptions	34.59%
Seq-to-Seq with BLSTM+Softmax	36×128 dsift descriptions	34.33%
	64×128 dsift descriptions	34.59%

**Table 7:** The accuracy rates of the RAF Compound test set based on model ensembling between ResNet50 trained on spatial domain images and models trained on densesift descriptions.

MergedModel	Inputs	TestAccuracy
ResNet50	Gray images	
DSAE+Softmax	4608 dsift descriptions	66.97%
ResNet50	Gray images	
LSTM	64×128 dsift descriptions	65.93%
ResNet50	Gray images	
BLSTM	36×128 dsift descriptions	66.32%
ResNet50	Gary images	
Seq-to-Seq with LSTM+Softmax	64×128 dsift descriptions	65.03%
ResNet50	Gary images	
Seq-to-Seq with BLSTM+Softmax	64×128 dsift descriptions	64.77%

**Table 8:** The accuracy rates of the RAF Compound test set based on model ensembling between ResNet50 trained on spatial and frequency domain images and models trained on densesift descriptions.

MergedModel	Inputs	TestAccuracy
ResNet50	Gray+WAVEDEC2_rbiosharpen	
DSAE+Softmax	4608 dsift descriptions	66.19%
ResNet50	Gray+WAVEDEC2_rbiosharpen	
LSTM	64×128 dsift descriptions	65.93%
ResNet50	Gray+WAVEDEC2_rbiosharpen	
BLSTM	36×128 dsift descriptions	65.80%
ResNet50	Gray+WAVEDEC2_rbiosharpen	
Seq-to-Seq with LSTM+Softmax	64×128 dsift descriptions	66.19%
ResNet50	Gray+WAVEDEC2_rbiosharpen	
Seq-to-Seq with BLSTM+Softmax	64×128 dsift descriptions	66.32%

**Table 9:** The abbreviations of the above expression categories.

Expression Category	Abbreviation
Angrily Disgusted	AD
Angrily Surprised	AS
Disgustedly Surprised	DS
Fearfully Angry	FA
Fearfully Surprised	FS
Happily Disgusted	HD
Happily Surprised	HS
Sadly Angry	SA
Sadly Disgusted	SD
Sadly Fearful	SF
Sadly Surprised	SS

**Table 10:** Comparison with several traditional classic operators on the RAF Compound test set.

Other Methods	Linear Kernel	RBF Kernel
HoG [38]+SVM	39.38%	40.28%
LBP+SVM	26.94%	28.24%
LGC [34]+SVM	20.73%	21.89%
HoG+LBP+SVM	39.12%	40.54%
HoG+LGC+SVM	38.86%	39.38%
HoG+LBP+LGC+SVM	37.56%	39.51%
ours (66.97%)	-	-

## Abbreviations

The following abbreviations are used in this manuscript:

DSAE	Deep Stacked AutoEncoder
MTCNN	Multi-task Cascaded Convolutional Neural Networks
LSTM	Long Short Term Memory
BLSTM	Bidirectional Long Short Term Memory
Seq-to-Seq	Sequence-to-Sequence
HE	Histogram Equalization
CLAHE	Contrast Limited Adaptive Histogram Equalization
DoG	Difference of Gaussian
FER2013	Facial Expression Recognition 2013
RAF Basic	Real World Affective Faces-Basic emotions
RAF Compound	Real World Affective Faces-Compound emotions
LGC	Local Gradient Code
HoG	Histogram of Gradient
LBP	Local Binary Pattern
SVM	Support Vector Machine
dsift	densesift
GNN	Graph Neural Network
GCN	Graph Convolutional Neural Network
